# Osteoblasts stimulate the osteogenic and metastatic progression of castration-resistant prostate cancer in a novel model for in vitro and in vivo studies

**DOI:** 10.1007/s10585-013-9626-1

**Published:** 2013-12-01

**Authors:** Malin Hagberg Thulin, Karin Jennbacken, Jan-Erik Damber, Karin Welén

**Affiliations:** Department of Urology, Sahlgrenska Cancer Center, Institute of Clinical Sciences, Sahlgrenska Academy at the University of Gothenburg, Box 425, 405 30 Göteborg, Sweden

**Keywords:** Castration-resistant prostate cancer, Bone metastasis, Osteoblasts, Osteomimicry

## Abstract

Castration-resistant prostate cancer (CRPC) is strongly associated with sclerotic bone metastases and poor prognosis. Models that mimic human CRPC are needed to identify the mechanisms for prostate cancer (PC) growth in bone and to develop new therapeutic strategies. We characterize a new model, LNCaP-19, and investigate the interaction between tumor cells and osteoblasts in the sclerotic tumor response of CRPC. Osteogenic profiling of PC cell lines (LNCaP-19, LNCaP, C4-2B_4_, and PC-3) was performed by gene expression arrays and mineral staining. Conditioned medium from MC3T3-E1 was used for osteoblast stimulation of CRPC cells. The capacity of LNCaP-19 cells to induce sclerotic lesions was assessed in intratibial xenografts and verified by serum markers, histological analysis and bone mineral density (BMD) measurements. The CRPC cell line LNCaP-19 expresses a pronounced osteogenic profile compared to its parental androgen-dependent cell line LNCaP. Osteoblast-derived factors further increase the expression of genes known to enhance metastatic progression of PC. LNCaP-19 forms sclerotic tumors in tibia of castrated mice as evident by increased total BMD (*P* < 0.01). There was a strong correlation between serum osteocalcin and BMD (total: *R* ^*2*^ 0.811, *P* < 0.01, trabecular: *R* ^*2*^ 0.673, *P* < 0.05). For the first time we demonstrate that a CRPC cell line generated in vitro has osteogenic capacity and that osteomimicry can be an inherent feature of these cells. Osteoblast-derived factors further promote the osteogenic and metastatic phenotype in CRPC cells. Altogether, our model demonstrates that both tumor cells and osteoblasts are mediators of the bone forming process of CRPC.

## Background

Today there are limited therapeutic alternatives for patients with advanced prostate cancer (PC), and androgen deprivation therapy (ADT) is the primary option. ADT is initially highly effective in suppressing tumor growth. However, it leads to increased bone degradation resulting in the release of growth factors from the bone matrix, many of which can stimulate prostate cancer (PC) cell attraction and tumor growth in bone [1–3]. The outcome for most patients with ADT-treated advanced PC is relapse of castration-resistant disease (CRPC) with bone metastasis, for which no curative treatment exists.

Bone metastases behave differently depending on their tumor origin. Metastases from breast and lung cancers are primarily osteolytic, inducing bone degradation [2, 4, 5], while bone metastases from PC form sclerotic lesions characterized by excessive deposition of unstructured new woven bone [3, 6–8]. These observations suggest that osteoblasts play a central role in the metastatic process in CRPC in the bone compartment.

Skeletal metastasis is the consequence of the bidirectional interplay between tumor cells and bone cells. Metastatic PC cells produce a variety of bone-stimulating factors including prostate specific antigen (PSA), endothelin-1, bone morphogenetic proteins (BMPs) and insulin-like growth factor 1 (IGF-1), that have direct or indirect effects on bone formation [9–11]. Moreover, osteoblasts secrete several factors including IGF-1, TGF-β and BMPs, which that facilitate the growth and survival of PC cells in the bone [12, 13]. These soluble factors potentiate a paracrine crosstalk between PC cells and bone cells, commonly termed “the vicious cycle” [2, 14] and play an important role in the formation of osteoblastic tumors in CRPC.

Tumor cells that preferentially metastasize to bone acquire features normally expressed by bone cells, particular to the osteoblastic phenotype, an adaptation referred to as osteomimicry [15–17]. The acquisition of osteogenic properties by tumor cells may enhance their capacity to communicate with their host organ and hence promote their capacity to survive and proliferate in the bone environment. It is not clear when the osteomimetic adaptation occurs. Some tumor cells at the primary site may already express a bone-like phenotype which would facilitate their metastasis to bone. Alternatively, seeding in the bone is stimulated by local factors converting the tumor cells to an osteomimetic stage (reviewed in [17]). The development of phenotypic bone changes in PC cells accompanying the development of castration resistance and communication with the bone environment is not well understood.

The understanding of the bone metastatic disease, and hence, the development of efficient and specific treatments is limited due to the difficulty of obtaining samples from bone metastases from patients. Therefore, models that mimic the human disease are critical for elucidation of alterations in both tumors and bone cells and for evaluation of new therapeutics. Today, few models exist to study the osteoblastic function of CRPC, for example LuCAP-23.1 and MDA-PCa 2a and 2b form osteoblastic lesions while C4-2B, IGR-CaP1 and PCSD1 give mixed osteolytic/osteoblastic lesions [18–22].

The overall aim with this study was to characterize a novel model, LNCaP-19, for studies of the osteoblastic process in metastases of CRPC under castrated conditions reflecting the clinical situation. LNCaP-19 was established in our laboratory as an in vitro derived castration-resistant subclone of LNCaP [23]. It has an increased angiogenic and invasive potential compared to its parental cell line [24, 25]. Compared to LNCaP, it also has metastatic potential [26, 27].

We here show that LNCaP-19 is suitable for studies of the osteoblastic function of CRPC, in a bidirectional system both in vitro and in vivo. We demonstrate that osteomimicry can be an inherent feature of PC cells, which is further potentiated by the acquisition of castration-resistance. We also show that osteoblasts trigger a more pronounced osteogenic and aggressive phenotype of the CRPC cells and initiate a cross-talk between tumor- and bone cells. Importantly, this study demonstrates that both tumor cells and osteoblasts are potential mediators of bone formation.

## Results

### LNCaP-19 has the capacity to mineralize extracellular bone matrix

**Fig. 1:**
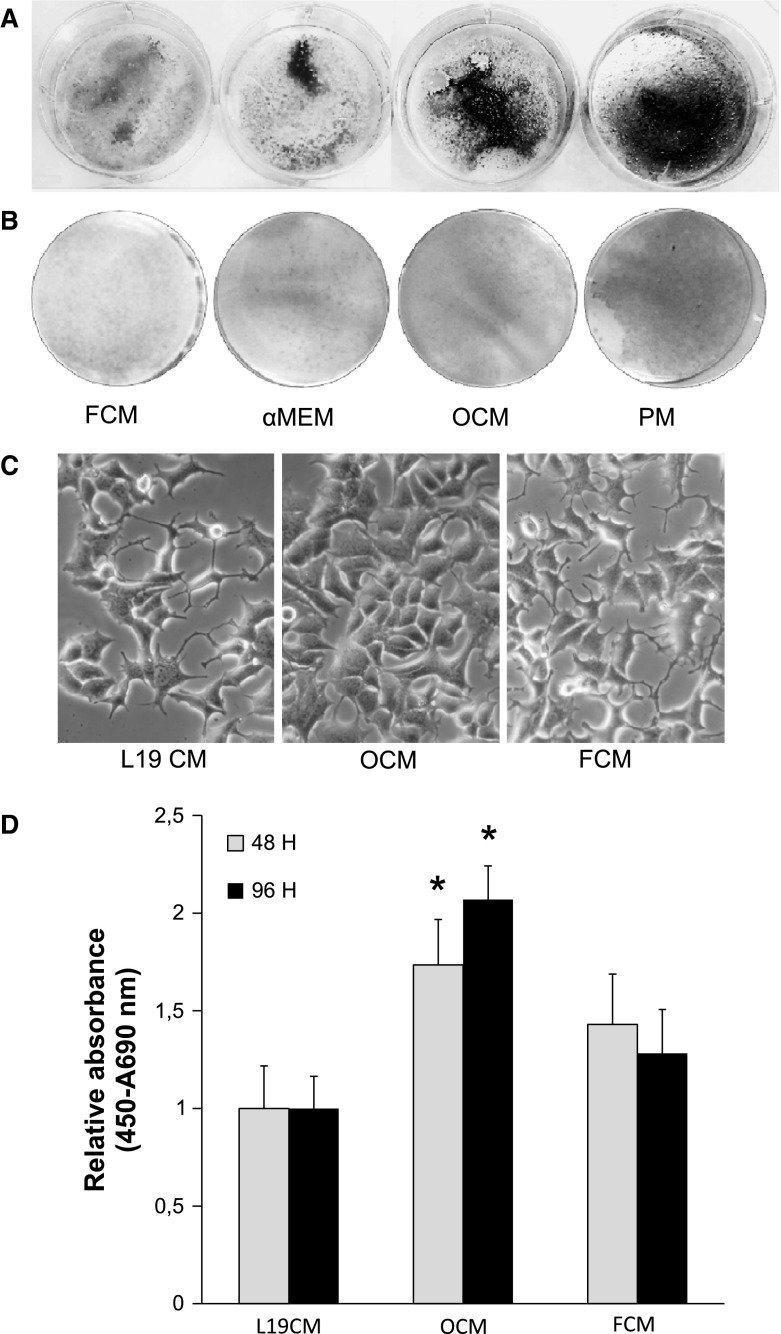
Osteoblasts induce LNCaP-19 mineralization and proliferation. LNCaP-19 cells were seeded in 6-well plates (1 × 10^5 ^cells/well) and in 12-well plates (4 × 10^4^ cells/well) for von Kossa staining respectively alkaline phosphatase (ALP) staining and cultured with fibroblast-conditioned medium (FCM), control medium (αMEM), osteoblast-conditioned (OCM), or promineralization medium (PM; αMEM supplemented with ascorbic acid (50 μg/ml) and β-glycerophosphate) (10 mM) under steroid deprived conditions (10 % FBS-DCC). **a** Mineralization of LNCaP-19, visualized after 21 days by von Kossa staining. **b** Staining of ALP activity after 21 days in culture. **c** Morphological changes in LNCaP-19 after 48 h in culture with OCM, FCM or control medium (L19CM) visualized with phase contrast light microscopy, magnification ×200. **d** Proliferation of LNCaP-19 cells. For proliferation, LNCaP-19 cells were seeded in 96-well plates (5 × 10^3^ cells/well) and stimulated with control medium (L19 CM), OCM or FCM. All media was supplemented with 1 % FBS-DCC. Proliferation of LNCaP-19 was measured by BrdU incorporation after 48 and 96 h.* Bars* represent mean ± SEM of three independent experiments. *P < 0.05 versus control

It is known that bone metastases are a result of the communication between bone and cancer cells and that PC cells acquire bone-like properties during disease progression. Therefore, in the present study, we investigated the ability of the LNCaP-19 cells to mineralize extracellular bone matrix. After 21 days of culture in steroid-depleted medium, both promineralization medium (PM) and osteoblast-conditioned medium (OCM) induced mineralization in LNCaP-19 compared to control medium (αMEM) or fibroblast-conditioned medium (FCM) (Fig. 1a). A strong staining of alkaline phosphatase (Alp) activity was detected in LNCaP-19 cells stimulated with PM, compared to cells cultured in control medium or OCM. FCM did not stimulate Alp activity (Fig. 1b). In agreement with a previous study [28], LNCaP did not mineralize when cultured in PM medium or in OCM (data not shown). Functional studies of the osteoblastic influence on LNCaP-19 cells demonstrated clear morphological changes in OCM stimulated cells compared to control cells (Fig. 1c). As shown in Fig. 1d, proliferation of OCM-stimulated LNCaP-19 cells was significantly increased (*P* < 0.05) compared to control cells treated with L19CM and FCM. This OCM-specific stimulation was only seen in LNCaP-19 since both OCM and FCM stimulated LNCaP proliferation 1.8 times compared to LNCaP-CM (data not shown).

### LNCaP-19 cells display a pronounced osteogenic gene expression profile

As the castration-resistant LNCaP-19 was able to mineralize, an osteogenesis gene signature array was used to identify the underlying gene program responsible for its osteomimetic properties. The osteogenic phenotype was compared between four different PC cell lines, LNCaP-19, mixed osteoblastic/osteolytic LNCaP (androgen-dependent) and C4-2B_4_ (castration-resistant) and the osteolytic castration-resistant cell line PC-3. All four cell lines expressed detectable levels of the majority of the genes included in the osteogenesis array, such as the transcription factor of the osteoblastic lineage msh homeobox 2 (*MSX2*) as well as bone morphogenetic protein (*BMP*)-*1*, -*4* and -*6*, *SMADs* and osteonectin (*Osn*, *SPARC*) (Table 1). The key transcription factor of the osteoblastic lineage runt-related transcription factor 2 (*RUNX2*) and alkaline phosphatase (*ALPL*) were expressed in LNCaP-19, LNCaP and PC-3 but absent in C4-2B_4_.

**Table 1:** Basal expression of osteogenesis- associated genes in prostate cancer cell lines

Gene symbol	Expression levels				Gene description
	LNCaP-19	LNCaP	C4-2B_4_	PC-3	
*AHSG*	ND	ND	*	ND	Alpha-2-HS-glycoprotein
*ARSE*	ND	ND	ND	**	Arylsulfatase E (chondrodysplasia punctata 1)
*ALPL*	**	*	ND	*	Alkaline phosphatase, liver/bone/kidney
*BGLAP*	ND	*	*	*	Bone gamma-carboxyglutamate (gla) protein
*BMP1*	***	**	**	**	Bone morphogenetic protein 1
*BMP2*	**	ND	ND	*	Bone morphogenetic protein 2
*BMP3*	ND	ND	ND	***	Bone morphogenetic protein 3
*BMP4*	**	**	**	***	Bone morphogenetic protein 4
*BMP5*	ND	*	ND	*	Bone morphogenetic protein 5
*BMP6*	**	**	**	**	Bone morphogenetic protein 6
*BMP7*	**	*	***	ND	Bone morphogenetic protein 7
*BMPR1A*	****	***	***	***	Bone morphogenetic protein receptor, type IA
*CDH11*	*	ND	ND	****	Cadherin 11, type 2, OB-cadherin
*COL1A1*	**	ND	ND	***	Collagen, type I, alpha 1
*COL3A1*	**	**	**	**	Collagen, type III, alpha 1
*COL4A3*	ND	ND	*	ND	Collagen, type IV, alpha 3
*COL4A4*	ND	ND	*	ND	Collagen, type IV, alpha 4
*COL4A5*	***	***	***	***	Collagen, type IV, alpha 5
*COL5A1*	**	**	*	***	Collagen, type V, alpha 1
*COL7A1*	****	*	**	**	Collagen, type VII, alpha 1
*COL9A2*	ND	**	**	*	Collagen, type IX, alpha 2
*COL12A1*	****	**	***	****	Collagen, type XII, alpha 1
*COL15A1*	ND	ND	ND	*	Collagen, type XV alpha 1
*COL16A1*	***	*	**	ND	Collagen, type XVI, alpha 1
*COL17A1*	ND	**	*	**	Collagen, type XVII, alpha 1
*COL18A1*	**	**	**	***	Collagen, type XVIII, alpha 1
*CSF2*	ND	ND	ND	*	Colony stimulating factor 2
*CSF3*	ND	ND	ND	**	Colony stimulating factor 3
*COMP*	**	*	**	*	Cartilage oligomeric matrix protein
*DSPP*	*	ND	*	ND	Dentin sialophosphoprotein
*EGF*	****	***	**	**	Epidermal growth factor
*EGFR*	****	***	***	****	Epidermal growth factor receptor
*ENAM*	ND	*	ND	*	Enamelin
*FGF1*	ND	*	ND	**	Fibroblast growth factor 1 (acidic)
*FGF2*	ND	*	ND	***	Fibroblast growth factor 2 (basic)
*FGFR1*	***	***	***	***	Fibroblast growth factor receptor 1
*FGFR2*	****	**	**	*	Fibroblast growth factor receptor 2
*FGFR3*	ND	*	*	ND	Fibroblast growth factor receptor 3
*IGF1*	ND	**	**	ND	Insulin-like growth factor 1
*IGF1R*	***	***	***	****	Insulin-like growth factor 1 receptor
*IGF2*	***	**	**	***	Insulin-like growth factor 2
*MGP*	**	*	***	*	Matrix Gla protein
*MINPP1*	****	***	***	***	Multiple inositol polyphosph. hist. phosphatase, 1
*MMP2*	**	ND	ND	*	Matrix metallopeptidase 2
*MMP13*	**	**	**	***	Matrix metallopeptidase 13 (collagenase 3)
*MSX1*	**	**	*	*	Msh homeobox 1
*MSX2*	**	**	***	**	Msh homeobox 2
*PDGFA*	****	***	***	***	Platelet-derived growth factor alpha polypeptide
*PHEX*	**	*	**	***	Phosph regulating endopeptidase homol. X-linked
*RUNX2*	**	*	ND	***	Runt-related transcription factor 2
*SMAD1*	****	***	***	***	SMAD family member 1
*SMAD2*	****	***	***	****	SMAD family member 2
*SMAD3*	***	***	**	****	SMAD family member 3
*SMAD4*	****	***	****	****	SMAD family member 4
*SMAD5*	****	****	****	****	SMAD family member 5
*SMAD6*	***	*	***	***	SMAD family member 6
*SMAD7*	****	***	***	**	SMAD family member 7
*SMAD9*	****	***	***	***	SMAD family member 9
*SOX9*	***	***	***	****	SRY (sex determining region Y)-box 9
*SPARC*	**	*	*	****	Secreted protein acidic, cysteine-rich (osteonectin)
*SPP1*	***	ND	ND	*	Secreted phosphoprotein 1 (osteopontin)
*TFIP11*	****	***	***	***	Tuftelin interacting protein 11
*TGFB1*	***	**	**	****	Transforming growth factor, beta 1
*TGFB2*	ND	**	**	***	Transforming growth factor, beta 2
*TGFB3*	***	*	**	***	Transforming growth factor, beta 3
*TGFBR1*	****	***	***	***	Transforming growth factor, beta receptor 1
*TGFBR2*	**	**	**	****	Transforming growth factor, beta receptor II
*TUFT1*	****	***	***	***	Tuftelin 1
*TWIST1*	***	**	ND	**	Twist homolog 1 (Drosophila)
*TWIST2*	ND	ND	ND	*	Twist homolog 2 (Drosophila)
*VDR*	***	***	***	***	Vitamin D (1,25-dihydroxyvitamin D3) receptor
*VEGFA*	****	***	***	***	Vascular endothelial growth factor A
*VEGFB*	****	***	***	***	Vascular endothelial growth factor B
*VEGFC*	ND	ND	ND	***	Vascular endothelial growth factor C

Gene expression in LNCaP-19, LNCaP, C4-2B4 and PC-3 cells after 48 h cultured under steroid deprived basal conditions analyzed by a gene signature array comprising 96 genes associated with osteogenesis. GUSB was used as endogenous control. The expression is graded based on Δ*Ct*-values (*Ct*-_*gene*_ − *Ct*-_GUSB_). **** Δ*Ct* < 1; *** Δ*Ct* < 5; ** Δ*Ct* < 10; * Δ*Ct* < 15. Δ*Ct* represents the mean value of three biological replicates. Non detected (ND) genes in LNCaP-19, LNCaP, C4-2B_4_, and PC-3 were, *AMBN*, *AMELY*, *CALCR*, *CASR*, *COL14A1*, *COL19A1*, *COL1A2*, *DMP1*, *FGF3*, *FLT1*, *GDF10*, *IBSP*, *MMP8*, *SOST*, *STATH*

LNCaP-19 and C4-2B_4_ are both derived from LNCaP and display osteogenic phenotypes. Comparing the osteogenic potential between these cell lines, LNCaP-19 exclusively has a basal expression of osteoblast-cadherin (*CDH11*), collagen 1a1 (*COL1A1*) and osteopontin (*Opn*, *SPP1*), all vital genes for tumor interaction with osteoblasts and osteoblastic activity. Despite the different status in androgen dependence, LNCaP and C4-2B_4_ share a similar gene expression pattern, such as the basal expression of osteocalcin (*Ocn*, *BGLAP*), fibroblast growth factor receptor (*FGFR*), insulin-like growth factor 1 (*IGF1*) and transforming growth factor, beta 2 (*TGFB2*). However, LNCaP-19 and C4-2B_4_ both have a basal expression of dentin sialophoshoprotein (*DSPP*), a gene associated with aggressive PC and mineralization process of enamel, which was absent in LNCaP.

A distinct pattern of genes involved in osteoclastogenesis and inhibition of bone formation, including colony stimulating factor 2 (*CSF2*), twist homolog 2 (*TWIST2*) and *BMP3*, were exclusively expressed in PC-3.

### Osteoblasts stimulate changes in the gene expression profile of prostate cancer cells

The osteogenesis gene signature array was further used to detect differently expressed genes in LNCaP-19, LNCaP, C4-2B_4_ and PC-3 cells after OCM-stimulation. Genes expressed at significantly different levels are presented in Table 2. Upon OCM stimulation, elevated expression of several genes associated with osteoblast function and tumor aggressiveness was demonstrated in both LNCaP-19 and LNCaP, including *DSPP* and *RUNX2.* A more pronounced osteogenic profile was observed in OCM stimulated LNCaP-19 cells compared to LNCaP, with increased expression of *CDH11* and matrix metalloproteinase-2 (*MMP2*) both associated with osteoblast communication and bone-tropic metastasis of PC. These genes were undetected in LNCaP. Furthermore, two genes were downregulated in LNCaP-19, collagen 16a1 (*COL16A1*) and matrix metalloproteinase-13 (*MMP13*; ~2 fold). Importantly, these genes are associated with chondrocytogenesis and osteoclastogenesis, respectively. The expression of the bone matrix proteins Osn and Opn was shown to be upregulated OCM-stimulated LNCaP cells, while their expression levels were unchanged in LNCaP-19. C4-2B_4_ was less responsive to OCM stimulation. However, C4-2B_4_, which lacks detectable basal expression of *RUNX2*, responded to OCM with induction of this transcription factor. Unique for C4-2B_4_ was the induction of fibroblast growth factor 1 and -2 after OCM stimulation.

**Table 2:** Differently expressed genes in prostate cancer cell lines after osteoblast stimulation

Gene symbol	Fold change				Gene description
	LNCaP-19	LNCaP	C4-2B_4_	PC-3	
*DSPP*	4.54*	+	=	=	Dentin sialophosphoprotein
*RUNX2*	3.82*	3.13	+	=	Runt-related transcription factor 2
*CDH11*	3.59*	ND	ND	=	Cadherin 11, type 2, osteoblast-cadherin
*COL3A1*	2.62	=	=	=	Collagen, type III, alpha 1
*MMP2*	2.43*	ND	=	=	Matrix metallopeptidase 2
*COL17A1*	+	=	=	=	Collagen, type XVII, alpha 1
*MMP13*	−2.22*	ND	=	=	Matrix metallopeptidase 13
*COL16A1*	−3.2	=	=	=	Collagen, type XVI, alpha 1
*SPARC*	=	3.47*	ND	=	Osteonectin
*SPP1*	=	+	ND	=	Osteopontin
*FGF1*	=	=	+	=	Fibroblast growth factor 1
*FGF2*	=	=	+	=	Fibroblast growth factor 2
*BMP2*	=	=	=	6.62*	Bone morphogenetic protein 2
*COL1A2*	=	=	=	+	Collagen, type I, alpha 2
*MGP*	=	=	=	−2.00	Matrix Gla protein
*ALPL*	=	=	=	−2.08	Alkaline phosphatase, liver/bone/kidney

mRNA expression in LNCaP-19, LNCaP, C4-2B_4_ and PC-3 was analyzed by an osteogenesis gene signature array after 48 h of OCM (osteoblast-conditioned medium) stimulation. The table shows genes that are up- or downregulated more than twofold. ND not detected, + only detected in OCM stimulated cells, = unchanged expression. Calculations on fold change are based on the ΔΔ*Ct* method on three independent replicates. *P < 0.05

None of the osteoblast-associated genes were changed in the osteolytic PC-3 cells in response to OCM stimulation. In contrast, PC-3 cells displayed an increased expression level of *BMP2*, an inhibitor of bone formation. In addition, significant downregulation of the Bmp2 antagonist *MGP* (matrix Gla protein) levels were demonstrated in OCM-stimulated PC-3 cells.

### LNCaP-19 stimulates osteoblast proliferation and mineralization

**Fig. 2:**
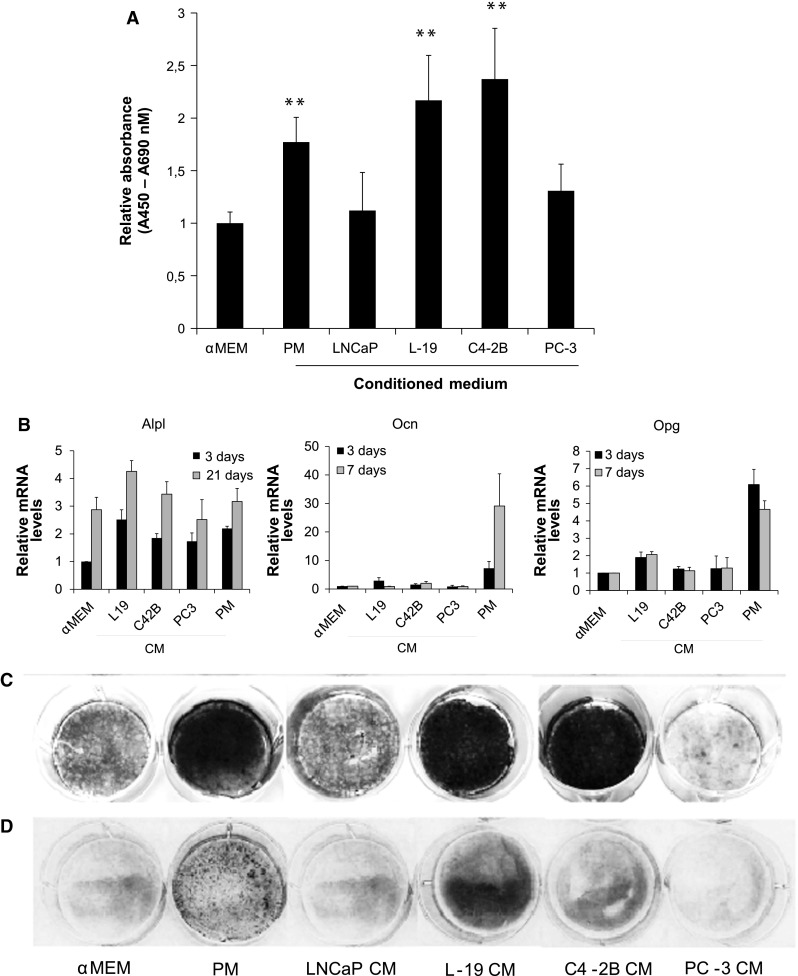
LNCaP-19 cells affect osteoblast proliferation and mineralization. **a** Proliferation of MC3T3-E1 (3.5 × 10^3^ cells per well in 96 well plates) was measured by BrdU incorporation after 5 days in culture with αMEM supplemented with 75 % CM. Bars represent mean ± SEM of three independent experiments. **P < 0.01 versus control medium (αMEM). Total RNA was extracted from MC3T3-E1 pre-osteoblasts at specified days and analyzed for the expression of differentiation markers by RT-qPCR. **b** *Alp1* day 3, and 21, *Ocn* at day 3 and 7, and *Opg* at day 3 and 7. The expression levels were normalized to those in control medium (αMEM). Results are presented as mean ± SEM of three independent experiments. **c** For mineralization assays, MC3T3-E1 pre-osteoblasts were seeded in 6-well plates (1 × 10^5^ cells/well) and in 12-well plates (4 × 10^4^ cells/well), for von Kossa staining respectively alkaline phosphatase (Alp) staining. Cells were treated with control medium (αMEM), conditioned medium (CM) from PC cell lines, or promineralization medium (PM; αMEM supplemented with ascorbic acid (50 μg/ml) and β-glycerophosphate) (10 mM). All media were supplemented with 10 % FBS. Mineralization of MC3T3-E1 was visualized using von Kossa staining after 21 days in culture. **d** Staining of Alp activity detected after 21 days in culture

To evaluate the effect of soluble factors from LNCaP-19 on the osteoblastic function, pre-osteoblasts (MC3T3-E1) were stimulated with conditioned medium (CM) derived from CRPC cell lines. The osteoblastic differentiation process is well characterized and consists of distinct phases of proliferation, matrix maturation and mineralization. CM with soluble factors derived from LNCaP-19 and C4-2B_4_ significantly (*P* < 0.01) induced proliferation of pre-osteoblasts to the same extent as PM treated cells (*P* < 0.01), while CM derived from PC-3 and LNCaP had no significant effect on pre-osteoblast proliferation comparable to αMEM control medium (Fig. 2a).

To further assess the effect of CRPC-secreted factors on osteoblastic differentiation, we studied the induction of mRNA expression of established differentiation markers in MC3T3-E1 pre-osteoblasts cultured with CM (Fig. 2b). Factors secreted from LNCaP-19 induced the expression of *Alpl*, which was sustained over 21 days, a pattern similar to C4-2B_4_. LNCaP-19 also induced *Ocn* expression at day 3 (~2.9 fold) compared to αMEM treated control cells, but the mRNA expression declined to control levels at day 7. Expression of endogenous osteoprotegerin (*Opg*), an inhibitor of osteoclast activation, was elevated by LNCaP-19 stimulation at day 3 and sustained upregulated at day 7. The main transcription factors of the osteoblastic lineage, *Runx2*, *Osx* and *Msx2* were not affected in MC3T3-E1 by CM stimulation (data not shown). In a control experiment, NIH3T3-E1 fibroblasts did not induce any of the osteoblast differentiation markers in response to LNCaP-19 CM (data not shown).

As shown in Fig. 2c, mineralization in osteoblasts was induced by the osteogenic CRPC cell lines, LNCaP-19 and C4-2B_4_, while the osteolytic PC-3 and LNCaP did not induce mineralization in osteoblasts. The observed *Alp* mRNA expression levels (Fig. 2b) and mineralization patterns of osteoblasts (Fig. 2c) were confirmed by staining of Alp activity (Fig. 2d).

### LNCaP-19 forms sclerotic lesions in mouse tibia

To evaluate the osteoblastic properties of LNCaP-19 in bone, LNCaP-19 cells were injected directly into bone marrow of tibia in castrated and non-castrated male BALB/c nude mice. After 10 weeks, 9 out of 13 tibiae of the castrated group and 8 of 13 in the non-castrated group developed tumors. Eight of the castrated and all of the non-castrated mice had tumors that were sclerotic with pronounced ossification.

**Fig. 3:**
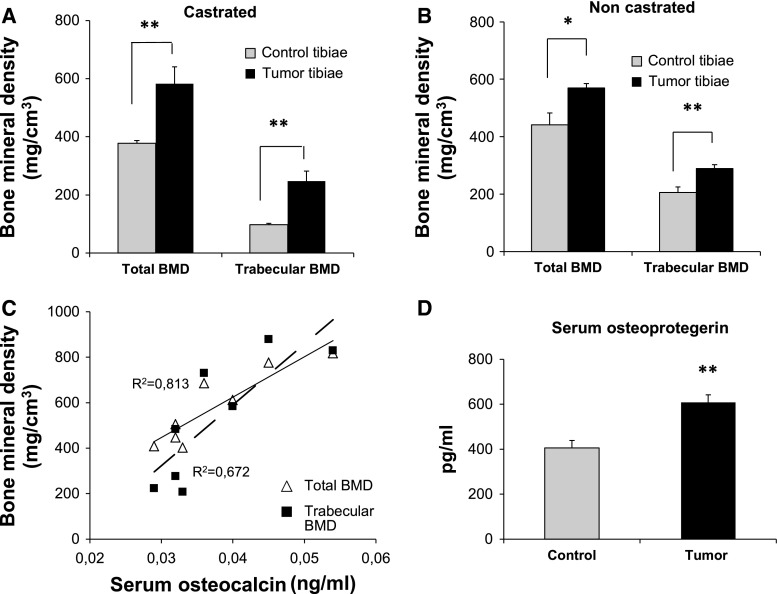
In vivo properties of LNCaP-19 evaluated as intratibial xenografts after 10 weeks of growth. LNCaP-19 cells (4 × 10^5^) were inoculated into tibia of castrated and non-castrated nude BALB/c mice. The osteosclerotic tumor response was evaluated after 10 weeks. **a**, **b** pQCT measurements demonstrate increased total and trabecular BMD in tumor-bearing tibiae compared to control tibiae in **a** castrated (*n* = 9) and **b** non-castrated (*n* = 8) mice. **c** Mouse-specific ELISA shows correlation between serum osteocalcin (Ocn) concentrations and BMD in castrated mice, for total BMD (*P* < 0.01) and trabecular BMD (*P* < 0.05), while no correlation was detected in non-castrated mice (data not shown). **d** The osteoprotegerin (Opg) levels as a response to LNCaP-19 tumors in both castrated and non-castrated tumor bearing mice (*n* = 8 + 9) were measured by mouse-specific ELISA and compared to control mice. *Bars* represent mean ± SEM. **P < 0.002

Increased bone formation was demonstrated by an increase in total bone mineral density (BMD) of the tumor-bearing tibiae compared to the non-tumor-bearing control tibiae in both castrated (*P* < 0.001) and non-castrated mice (*P* < 0.028). The sclerotic effect was also demonstrated with a pronounced difference of trabecular BMD to the control tibia, in the castrated group (*P* < 0.002) and the non-castrated (*P* < 0.003) (Fig. 3a, b). Ocn is a marker of osteoblast activity and is often elevated during increased bone turnover, for example after castration. The osteoblastic response of LNCaP-19 in castrated mice was also reflected in a strong correlation between serum levels of mouse Ocn and total BMD (*R* ^*2*^ 0.811, *P* < 0.01) as well as trabecular BMD (*R* ^*2*^ 0.673, *P* < 0.05) (Fig. 3c). There was no significant correlation between Ocn and BMD in the group of non-castrated mice (Total BMD: *R* ^*2*^ 0.220, trabecular BMD: *R* ^*2*^ 0.243) (data not shown). The sclerotic response in LNCaP-19 tumors may partly depend on inhibited osteoclast activation and osteolysis, since Opg serum level was significantly elevated in tumor bearing castrated and non-castrated mice compared to control mice (*P* < 0.002).

**Fig. 4:**
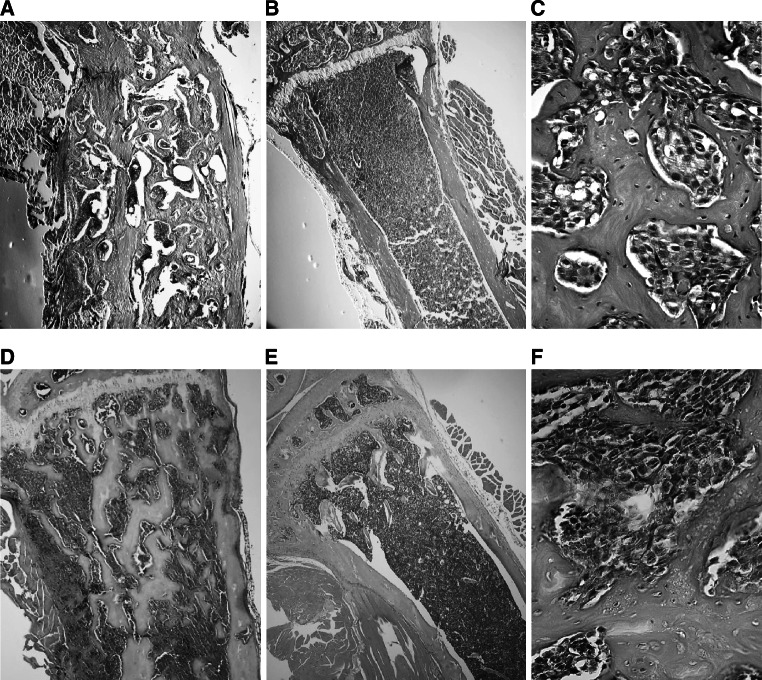
Histology of the sclerotic tumor response of LNCaP-19. LNCaP-19 cells (4 × 10^5^) were inoculated into tibia of castrated and non-castrated nude BALB/c mice. The osteosclerotic tumor response was evaluated after 10 weeks by histological evaluation of LNCaP-19 in tibiae of castrated and non-castrated mice. **a**, **d** Longitudinal sections (H&E staining) of LNCaP-19 in the tibia, demonstrating new formed bone replacing the bone marrow cavity (magnification ×200). **b**, **e** Control tibiae without tumor cell injection (magnification ×200). **c**, **f** Islands of interlaced LNCaP-19 cells within the newly formed trabecular bone (magnification ×400). **a**–**c** represent tibiae from castrated mice and **d**–**f** non-castrated

The sclerotic response was clearly demonstrated in the tumor-bearing tibiae of castrated mice with new formed bone replacing the bone marrow cavities, enforced cortex and mineralization in progress (Fig. 4a). An infiltrative growth of prostate carcinoma was demonstrated as interlaced islands of tumor cells within the irregular net of newly formed bone (Fig. 4c). A similar sclerotic response was demonstrated in non-castrated mice (Fig. 4d, f). The injection alone, without tumor cells, did not generate any increased BMD in a control experiment. The control tibiae in castrated mice displayed a total absence of trabecular bone (Fig. 4b) compared to control tibiae in the non-castrated group (Fig. 4e).

## Discussion

Experimental models are important for increased understanding of the complex interplay between tumor and bone cells in PC metastasis. In a new model for studies of osteoblastic tumors of CRPC, which closely mimic the clinical situation, we demonstrate that tumor cell stimulation by osteoblast-secreted factors is a contributing factor to the vicious cycle of sclerotic bone metastasis of PC.

Since it is known that PC cells acquire bone-like properties during disease progression we performed an osteogenic profiling of castration-resistant LNCaP-19 and its parental androgen-dependent cell line LNCaP. A basal expression of bone-related genes in favour of an osteoblastic response was detected in both cell lines, while expression of genes associated with osteolytic events and osteoclastogenesis was low or undetectable. However, there is a major difference between the two cell lines regarding the osteogenic properties; LNCaP-19 is able to mineralize extra cellular bone matrix whereas LNCaP lack this ability. In accordance with its capacity to mineralize, LNCaP-19 expresses genes such as *COL1A1* and *DSPP*, both crucial for the mineralization process, while their mRNA expression was undetectable in LNCaP cells. Therefore, our data suggest that the basal osteomimetic properties of LNCaP-19 are the result of the acquisition of castration-resistance and may explain part of the tropism of CRPC cells to bone.

It has previously been demonstrated that a number of proteins, such as Osn, Opn and Ocn, directly associated with bone growth and osteoblast activity are overexpressed in tumor cells during the transformation of PC cells into a castration-resistant state [16, 29, 30]. In accordance with those previous studies, and indicating the importance of castration-resistance in osteomimicry, these genes, except for Ocn, are highly expressed in LNCaP-19 as compared to LNCaP. However, the mRNA expression of Osn, Opn and Ocn was directly induced in LNCaP by osteoblast-stimulation, suggesting that osteoblasts can promote an osteogenic phenotype prior to castration-resistance.

To further characterize LNCaP-19 as an osteogenic CRPC model, we compared the bone-associated gene profile of LNCaP-19 with other CRPC cell lines. Compared to both the parental androgen-dependent LNCaP and the in vivo derived osteoblastic castration-resistant cell line C4-2B_4_, LNCaP-19 expressed several genes associated with osteoblastic properties that were not expressed in the other cell lines (*CDH11*, *COL1A1* and *SPP1*), which could explain its osteoblastic potential also in steroid deprived conditions. The absence of *RUNX2* expression in C4-2B_4_ in basal steroid deprived conditions is a striking difference, which could possibly be due to the fact that C4-2B_4_ was derived in vivo, from a bone metastasis from C4-2 [22]. Thus, the osteoblastic function of C4-2B_4_ in vivo may be dependent on the presence of bone cells to induce *RUNX2*, which was also demonstrated in vitro in the present study. In contrast, LNCaP-19, which is developed in vitro without influence from bone, has the full osteogenic gene programme activated independent of osteoblast stimulation.

The strongly osteolytic cell line PC-3 displayed most of the osteogenic profile but in contrast to LNCaP-19 also expressed a different set of genes strongly associated with osteoblast inhibitory and osteolytic events, such as *BMP3*, *TWIST2* and *CSF2* and -3. This observation suggests that the expression of these genes may prevent the ability of PC-3 to mineralize or to induce mineralization in osteoblasts. Moreover, the bone-forming genes that were observed to be elevated in response to osteoblast-derived factors in LNCaP-19 in comparison to PC-3 cells, suggesting that soluble osteoblast-derived factors increase the osteogenic potential of osteogenic CRPC cells, while the effect on osteolytic cells is minor.

The observation that LNCaP-19 has the capacity to mineralize matrix without osteoblast stimulation suggests that osteomimicry can be an inherent property in advanced PC, independent of bone stimulation. Interestingly, LNCaP-19 was derived from a lymph node metastasis and further progressed by androgen deprivation in vitro, and thus has never experienced contact with the bone environment. However, osteogenic capacity has previously been demonstrated in other CRPC cell lines (C4-2B_4_, MDA-PCa 2a and 2b) [19, 28], but in contrast to LNCaP-19 these cell lines are derived from bone implants and may therefore have adopted phenotypic changes in the bone milieu.

Osteoblast-secreted factors induced a phenotypic shift of LNCaP-19 cells, including morphological changes, increased proliferation and capacity to mineralize matrix. Interestingly, this phenotypic shift of LNCaP-19 is associated with elevated expression of genes related to tumor aggressiveness and metastasis of CRPC. Among these was *RUNX2*, the main transcription factor of osteoblast differentiation, which has previously been found to be ectopically expressed in metastatic cancer cells [31–33]. In PC, Runx2 expression increases with disease progression where it can induce an aggressive gene expression program, including normally weakly expressed genes such as *MMP2* [34, 35]. This indicates a different regulatory role of Runx2 when ectopically expressed in tumor cells. Moreover, it has been shown that mineralization of C4-2B_4_ was induced by Runx2 [28]. This suggests that *RUNX2* have an important role in the development of the osteogenic phenotype of CRPC. The relatively weak response of C4-2B_4_ to OCM stimulation may be a result of the conditions under which it was developed. The growth in bone may have selected for cells that were well adapted to that microenvironment, and as a result an additional OCM stimulation in vitro does not dramatically change its phenotype. However, in vitro OCM stimulation induced expression of fibroblast factor 1(*FGF1*) and -2(*FGF2*) exclusively in C4-2B_4_ cells. Since FGF1 has been shown to prevent osteoporosis in a rat model [36], this could suggest a different sclerotic mechanism in bone-derived CRPC cells compared to CRPC cells that have never been in contact with bone, such as LNCaP-19.

The vicious cycle involving tumor- and bone cells in osteolytic metastases from breast and lung cancer has been well characterized [37]. However, the vicious cycle of osteoblasts and PC tumor cells in the sclerotic situation is poorly understood. The upregulated expression of *CDH11* and *MMP2* in LNCaP-19 in response to OCM indicates induction of an increased potential for interaction between osteoblasts and PC cells in the formation of bone metastases [38, 39]. Recently, *MMP2* was identified as a key factor of the vicious cycle of osteoblasts and metastatic tumor cells [40]. The *CDH11*, also known as osteoblast-cadherin, is reported to be highly expressed in bone metastases and bone-derived PC cell lines. Moreover, *CDH11* was previously reported to mediate cell-invasion and adhesion between PC cells and osteoblasts [38, 39]. Alterations of *CDH11* are suggested to be partly responsible for the bone-tropism of PC. The expression of *MMP2 and CDH11* in LNCaP-19 and PC-3 in comparison to LNCaP and elevated expression levels of these genes upon OCM stimulation in LNCaP-19, suggests that castration-resistance potentiates the communication between tumor cells and osteoblasts.

In addition to osteoblast-induced changes in CRPC cells, we demonstrate that LNCaP-19 stimulates pre-osteoblasts to differentiate, proliferate and mineralize in a manner similar to other osteoblastic CRPC models [19, 28]. Previous studies have reported that stimulation by CRPC cells led to a significantly increase in an immature population of osteoblasts [28, 41]. In accordance with this, we demonstrated the effect of LNCaP-19 stimulation on osteoblasts including an early induction of *Ocn* on day 3 and maintained expression of *Alpl* over the differentiation process. Taken together, these findings are in agreement with clinical observations of CRPC metastases, which are composed of a high proportion of immature new woven bone and non-mineralized matrix [42], which is also clinically reflected by increasing Alp levels in serum accompanied by a modest increase of Ocn [43]. Ocn is a marker of mature osteoblasts and is normally induced at a later stage than Alp, which is necessary for the initiation but not for continuation of mineralization during osteoblast differentiation [44]. In line with previous observations, the altered pattern of differentiation markers expressed in osteoblasts accompanied by a different staining pattern of mineralization and increased proliferation, suggests that LNCaP-19 cells stimulate an immature population of osteoblasts that contributes to the major part of the newly synthesized bone. In addition, the positive correlation between serum Ocn and BMD in the in vivo system, supports evidence for increased osteoblast activity in response to LNCaP-19.

Osteoblasts can regulate bone metabolism by modulating the bone resorption process, directly via the production of receptor activator of nuclear factor κβ ligand (RANKL) and negatively via the secretion of its decoy receptor Opg [45, 46]. In the present study, the elevated mRNA expression of *Opg* and serum level of Opg in osteoblasts and tumor bearing mice respectively, in response to LNCaP-19, suggest a local suppression of osteoclast activation which could contribute to the overall osteoblastic response in CRPC tumors.

Furthermore, the present study demonstrates the capacity of LNCaP-19 to form sclerotic lesions in bone upon intratibial implantation in both castrated and non-castrated mice. Interestingly, LNCaP-19 was shown to induce an osteoblastic response in tibiae of castrated mice with increased BMD. This in contrasts to the commonly used LNCaP bone-derived C4-2 model, which under castrated conditions results in mixed osteoblastic/osteolytic lesions with an overall decrease in BMD [47]. We also demonstrate that in castrated mice, BMD positively correlate with Ocn serum levels, indicating additional tumor-induced osteoblast activity in the castrated compared to the non-castrated situation.

With similarity to clinical observations [42], the LNCaP-19 tumors in tibia show characteristics of an osteoblastic tumor response (i.e. osteodense bone without resorption). The newly formed bone was observed in the marrow spaces, not adjacent to the cortical bone surface associated with osteoclast-dependent bone resorption as occurs in the normal bone formation.The pattern of irregularly formed trabecular bone, interlaced with islands of tumor cells closely mimics a metastatic prostate adenocarcinoma in humans, rendering this model useful for in vivo evaluation.

In conclusion, our work presents a model, closely mimicking the clinical situation, for studies of the osteoblastic function of CRPC both in vitro and in vivo. We here show that osteomimicry can be an inherent feature of CRPC cells, which is further promoted by osteoblast stimulation. Moreover, osteoblast-secreted factors trigger the aggressiveness of CRPC cells and potentiate cross-talk between tumor- and bone cells in the sclerotic situation. Collectively these results indicate that targeting the communication between osteoblasts and PC cells could inhibit the progression of bone metastases in CRPC.

## Materials and methods

### Cell lines and culture conditions

The androgen-dependent PC cell line LNCaP, FGC clone, was obtained from the American Type Culture Collection (ATCC, Rockville, MD). LNCaP-19 a CRPC cell line, derived from LNCaP by androgen deprivation, was established in vitro in our laboratory [23]. PC-3 was obtained from the European Collection of Cell Cultures (ECCC, Wiltshire, UK). C4-2B_4_ was kindly provided by Professor Thalmann, Dept of Urology, University of Bern, Switzerland. PC cells were routinely maintained in RPMI-1640 supplemented with glucose, sodium pyruvate (PAA), 10 % foetal bovine serum (FBS, Invitrogen, Carlsbad, CA) (LNCaP, PC-3 and C4-2B_4_) or 10 % dextrane charcoal-treated FBS (FBS-DCC, Invitrogen) (LNCaP-19). Pre-osteoblasts, MC3T3-E1 cells, clone 4, were obtained from the ATCC (Rockville, MD). Osteoblasts were maintained in ascorbic acid (AA) free αMEM (Invitrogen) and 10 % FBS. Murine fibroblasts (NIH3T3, ATCC) were maintained in DMEM (Invitrogen) supplemented with 10 % FBS. All cultures were supplemented with penicillin/streptomycin. Passage numbers between 17 and 21 were used for LNCaP-19 cells and passage numbers 5–9 for PC-3 and C4-2B_4_. For osteoblasts and fibroblasts, passage numbers between 3 and 8 were used in the experiments. Passage number 1 is defined as the first passage in our laboratory.

To induce mineral production, cells were cultured in promineralization medium (PM) consisting of αMEM supplemented with 10 mM β-glycerophosphate (Sigma Chemical Co., St. Louis, MO, USA) and 50 mg/ml L-AA (Sigma) or cell-conditioned medium (CM) for 21 days.

### Conditioned medium

Cell-conditioned medium (CM) was obtained to assess the effects of secreted factors produced by osteoblasts and CRPC cells on gene expression level, proliferation and mineralization. Cells were seeded in T75 flasks and grown to 70–80 % confluence in their respective media, cells were washed with PBS and medium was replaced with serum-free αMEM. CM was collected after 48 h culture. The cell number was determined and CM was adjusted by dilution in serum-free medium to normalize for differences in cell density. CM was centrifuged to remove cell debris and stored at −20 °C until use. Maximal final concentration of CM used in the experiments was 75 %.

Abbreviations used: CM (CRPC-conditioned medium from LNCaP-19, C4-2B_4_, PC-3, and LNCaP), OCM (osteoblast-conditioned medium from MC3T3-E1) and FCM (fibroblast-conditioned medium from NIH3T3).

### Osteoblast differentiation

Osteoblasts were seeded in T25 flasks (3 × 10^5^) as described above. After 48 h, cells were washed with PBS and standard medium was replaced with αMEM, PM or CM. Cell culture medium for osteoblasts and fibroblasts was supplemented with 10 % FBS. For studies of osteoblastic differentiation markers, cultures were maintained in CM for 3, 7 or 21 days, when cells were harvested for RNA extraction. Medium was changed every 3 days.

### Cell proliferation

Osteoblasts were seeded in 96 well plates at a density of 3.5 × 10^3^ cells per well in αMEM with 10 % FBS in four replicates for each treatment. On day 2, medium was changed to CM, PM or control medium (αMEM). Since osteoblasts are dependent on serum for survival in culture, all medium was supplemented with 10 % FBS. LNCaP-19 cells were seeded at a density of 5.0 × 10^3 ^cells per well in αMEM with 1 % DCC. Medium was replaced on day two with OCM, FCM, or control medium (CM from LNCaP-19; L19CM), supplemented with 1 % FBS-DCC. Proliferation was monitored by the BrdU ELISA colorimetric assay (Roche Diagnostics, Indianapolis, IN) in accordance with the manufacturer’s instructions after 48, 96 h (LNCaP-19) and 5 days (MC3T3-E1). Stop solution (1 M H_2_SO_4_) was added and plates were read in a Wallac 1420 Victor3™ multiplate reader (PerkinElmer, Inc, Shelton, CT) at a 450 nm with reference filter at a 690 nm.

### Detection of mineralization with von Kossa and alkaline phosphatase staining

MC3T3-E1, LNCaP and LNCaP-19 cells were seeded in six well plates at a density of 1 × 10^5^ or 2 × 10^5^ cells per well in αMEM with 10 % FBS or RPMI-1640 with 10 % FBS-DCC, respectively. After 48 h, medium was changed to CM, OCM or PM and mineral deposition was determined by von Kossa or alkaline phosphatase staining after 21 days. Briefly, for von Kossa, cells were washed with PBS and fixed with 95 % ice cold ethanol. For calcium retention, the fixed cells were exposed to 60 W UV light in a solution of 5 % silver nitrate for 60 min for visualization. Alkaline phosphatase (Alp) is a marker of proliferating osteoblasts and is greatly enhanced during in vitro bone formation. In brief, MC3T3-E1 and LNCaP-19 cells were fixed for 60 s using neutral buffered formalin (10 %) and washed with PBS + 0, 05 TWEEN 20. BCIP/NBT (Sigma; 5-bromo-4-chloro-3-indolyl phosphate/nitro blue tetrazolium) substrate solution was added to the monolayer and incubated in dark for 25 min and washed before visualization. For the mineralization assays, medium was changed every 3 days.

### RNA isolation and RT-qPCR

Total RNA was extracted using RNeasy Mini Plus kit (Qiagen, Valencia, CA) in accordance with the manufacturer’s instructions. RNA concentration was measured on a NanoDrop (Thermo Fisher Scientific Inc, Wilmington, DE). A total of 1 μg RNA per reaction was reversely transcribed into cDNA using the VILO Superscript cDNA synthesis kit (Invitrogen) in accordance with the manufacturer’s instructions. RT-qPCR was performed using an ABI Prism 7500 Fast Sequence Detector (Applied Biosystems, Foster City, CA) and specific TaqMan probes as follows: mouse runt-related transcription factor 2 (*Runx2*) (*Cbfa1*;Mm00501584), osterix (*Osx*) (Mm00501584_m1), msh homeobox 2 (*Msx2*) (Mm00442992_m1), alkaline phosphatase (*Alp1*) (Mm01187117_m1) osteocalcin (*Ocn*) (*Bglap*;Mm03413826_m1), osteoprotegerin (*Opg*) (Mm01205928_m1) and endogenous control 18S rRNA (Hs99999901), purchased as TaqMan Gene Expression Assays (Applied Biosystems). PCR parameters were in accordance with the manufacturer’s protocol and the ΔΔ*Ct* method was used for relative mRNA quantification. The expression levels of each sample were normalized against 18S rRNA and PCR reactions for target genes, and controls were performed in triplicates for all samples.

### Osteogenesis gene signature array

Expression of bone-related genes in LNCaP-19, LNCaP and PC-3 were evaluated using a human osteogenesis gene signature array (Applied Biosystems: TaqMan^®^ Array Gene Signature Plate #4418741). The array included 96 osteogenesis-related genes and endogenous control genes (for a complete list of genes included, see www.appliedbiosystems.com). The array was used to evaluate the basal osteogenic expression profile of LNCaP-19, LNCaP, C4-2B_4_ and PC-3 with or without stimulation with OCM. Briefly, cells were seeded in T25 flasks in an optimized density (LNCaP and LNCaP-19, 1 × 10^6^, C4-2B, 8 × 10^5^ and PC-3, 6 × 10^5^) to reach 80 % confluence after 24 h. Cells were starved for 24 h before medium was replaced with control medium or OCM supplemented with 1 % FBS-DCC. Cultures were maintained for 48 h before RNA extraction, as previously described. A total of 5 μg cDNA/reaction was used for the PCR reactions. Parameters were set up in accordance with the manufacturer’s protocol and the ΔΔ*Ct* method was used for relative mRNA quantification. The expression levels of each sample were normalized against *HPRT1* (Hs99999909_m1) and *GUSB* (Hs99999908_m1). Only target genes with the most significant variations are shown (twofold regulation). Genes were considered as non-detected in cases where *Ct* values were above 36. Gene expression analysis was performed in independent triplicates.

### Xenograft model and bone impact analyses

An intrabone injection in vivo model was used to confirm the osteoblastic phenotype induced by LNCaP-19 in vitro. Eight-week-old male BALB/c nude mice (Charles River, Wilmington, MA) were castrated via scrotal incision under anaesthesia. LNCaP-19 cells were then injected into the left tibia using a procedure similar to what has previously been described [18, 27]. Briefly, the left leg of the mouse was flexed to a 90° angle and a 29 G needle was inserted, via the knee joint, into the proximal end of tibia using a drilling motion. 400,000 LNCaP-19 cells suspended in 7 μl matrigel (BD Bioscience, Franklin Lakes, NJ) were injected directly into the bone marrow cavity. Animals received analgesics (Rimadyl; 5 mg/kg) the following 5 days. The experiment was ended after 10 weeks. Blood was collected and stored as serum at −70 °C until analysis. Tibiae were dissected and bone mineral density (BMD) was obtained from CT scans of tibiae using pQCT XCT RESEARCH M (version 4.5B: Norland, Ft. Atkinson, WI). For each mouse, the right tibia was used as non-tumor-bearing control. For histological evaluation, tibiae were fixed in formalin, decalcified in modified formic acid (Histolab, Sweden) and embedded in paraffin. Tumor establishment was assessed in 4 μm thick hematoxylin and eosin (H&E) stained sections of the central part of the bone marrow.

Ethics statement: This study was carried out in strict accordance with the recommendations in the Guide for the Care and Use of Laboratory Animals of the National Institutes of Health. The protocol was approved by the local Committee on the Ethics of Animal Experiments of Gothenburg (Permit Number: 109-2008). All surgery was performed under anaesthesia, and all efforts were made to minimize suffering.

### ELISA

Serum levels of Ocn, a marker of osteoblast activity and Opg, an inhibitor of osteoclast activity, were measured using mouse-specific ELISAs in accordance with the manufacturer’s instructions (Ocn; Demeditec Diagnostics GmbH, Germany, Opg; Abcam, Cambridge, UK). Serum was diluted 1:10 and absorbance was read at 450 nm in a Wallac 1420 Victor3™ multiplate reader (PerkinElmer Inc, Shelton, CT).

### Statistics

Statistical calculations were performed using the SPSSv 20 software package (SPSS, Chicago, USA). Statistical differences between groups were performed using Student’s *t* Test or Mann–Whitney *U* Test where appropriate. Statistical significance of BMD was determined by Mann–Whitney *U* test. Correlation was determined with Spearman’s rank correlation test. All data are presented as mean ± SEM. A *P* value of < 0.05 was considered significant.
